# Emerging and Promising Multifunctional Nanomaterial for Textile Application Based on Graphitic Carbon Nitride Heterostructure Nanocomposites

**DOI:** 10.3390/nano13030408

**Published:** 2023-01-19

**Authors:** Dominika Glažar, Ivan Jerman, Brigita Tomšič, Raghuraj Singh Chouhan, Barbara Simončič

**Affiliations:** 1 Faculty of Natural Sciences and Engineering, University of Ljubljana, Aškerčeva Cesta 12, 1000 Ljubljana, Slovenia; 2 National Institute of Chemistry, Hajdrihova 19, 1000 Ljubljana, Slovenia; 3 Jožef Stefan Institute, Department of Environmental Sciences, Jamova Cesta 3, 1000 Ljubljana, Slovenia

**Keywords:** g-C_3_N_4_, Ag, TiO_2_, nanomaterial, heterostructures, textile, functionalization

## Abstract

Nanocomposites constructed with heterostructures of graphitic carbon nitride (g-C_3_N_4_), silver (Ag), and titanium dioxide (TiO_2_) have emerged as promising nanomaterials for various environmental, energy, and clinical applications. In the field of textiles, Ag and TiO_2_ are already recognized as essential nanomaterials for the chemical surface and bulk modification of various textile materials, but the application of composites with g-C_3_N_4_ as a green and visible-light-active photocatalyst has not yet been fully established. This review provides an overview of the construction of Ag/g-C_3_N_4_, TiO_2_/g-C_3_N_4_, and Ag/TiO_2_/g-C_3_N_4_ heterostructures; the mechanisms of their photocatalytic activity; and the application of photocatalytic textile platforms in the photochemical activation of organic synthesis, energy generation, and the removal of various organic pollutants from water. Future prospects for the functionalization of textiles using g-C_3_N_4_-containing heterostructures with Ag and TiO_2_ are highlighted.

## 1. Introduction

As a next-generation visible-light-active photocatalyst, graphitic carbon nitride (g-C_3_N_4_) has attracted considerable attention in various scientific fields, including environment remediation [1,2,3,4,5,6,7,8,9,10,11], energy storage and conversion [4,5,8,12,13,14,15,16,17], and biomedicine [18,19,20], which are the most important applications. It has already emerged as a promising nanomaterial for the degradation of various organic and inorganic environmental pollutants [1,2,3,4,5,6,8,9,10,11], CO_2_ reduction [3,8,15], NO_x_ removal [3], hydrogen evolution through water splitting [3,4,8,12,13,14,15,16], supercapacitors and batteries [5,8], solar and fuel cells [17], diagnostic imaging [19], therapeutic applications [19,20], biosensors [4,7,18,19,20], and antibacterial disinfection [3,19,20]. Accordingly, there have been a large number of scientific publications on g-C_3_N_4_ and g-C_3_N_4_ heterostructures, including more than 2000 original and review articles in 2022 alone (source: Web of Science, advanced search query preview: “graphitic carbon nitride or g-C_3_N_4_” and “photocataly*” in abstracts in 2022, assessed on 28 December 2022).

**Figure 1:**
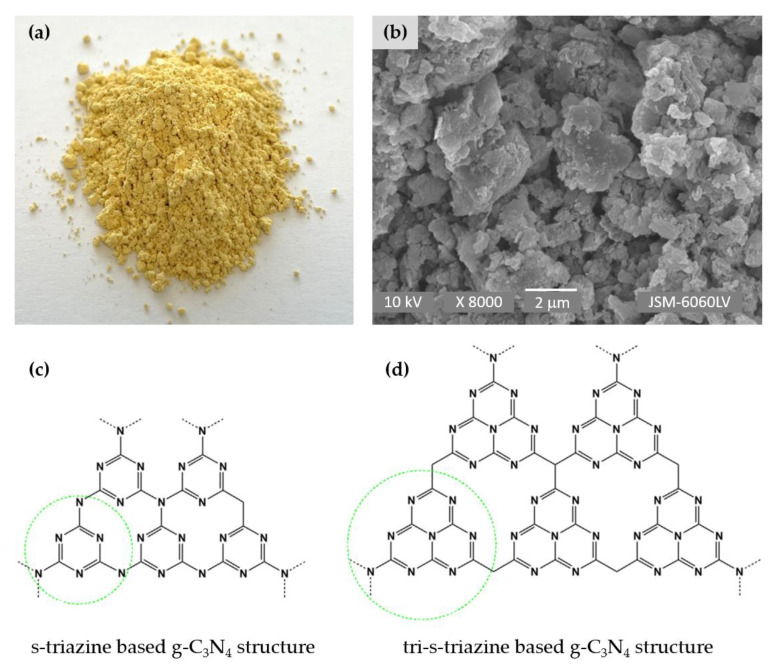
Schematic presentation of g-C_3_N_4_: photo of g-C_3_N_4_ powder (**a**), SEM image of g-C_3_N_4_ nanosheets (**b**), s-triazine (**c**), and tri-s-triazine (**d**) structure of g-C_3_N_4_. Reprinted with permission from [19]. Copyright 2020, Elsevier.

The attractiveness of g-C_3_N_4_ is directly related to its properties, as it is distinguished as a sustainable, organic, and metal-free two-dimensional conjugated polymeric n-type semiconductor with unique optical and electronic properties, high physicochemical and thermal stability, and high corrosion resistance, in addition to having a high earth abundance and an easy and inexpensive means of fabrication [21]. Due to its mild band gap of about 2.7 eV, g-C_3_N_4_ responds to visible light with an optical absorption edge of about 460 nm and, therefore, enables visible-light-driven photocatalytic reactions [22,23]. Pure g-C_3_N_4_ consists of carbon and nitrogen elements and is usually prepared through thermal polycondensation from nitrogen-rich precursors, such as melamine, urea, thiourea, dicyandiamide, cyanamide, and cyanuric acid, in the temperature range between 450 °C and 650 °C (Figure 1a,b) [8,22,24,25,26,27]. It has a graphite-like layered structure composed of aromatic s-triazine (C_3_N_4_) (Figure 1c) and tri-s-triazine (C_6_N_7_) (Figure 1d) rings linked by tertiary amines. The layers are held together by weak van der Waals forces. Since g-C_3_N_4_ synthesized in this way is bulky and exhibits low surface area, marginal optical absorption in the visible region, rapid charge recombination, and low charge mobility, various nanostructured forms of g-C_3_N_4_ in different morphologies with higher photocatalytic activity have been prepared, including 3D porous structures, 2D nanosheets, 1D nanorods/nanotubes, and 0D g-C_3_N_4_ quantum nanodots [4,7,21,25].

Another important strategy to improve the photocatalytic efficiency of g-C_3_N_4_ is the formation of g-C_3_N_4_-based binary and ternary heterostructure composites, including doping/loading with noble metals and creating heterojunctions with other organic and inorganic semiconductors [15,28]. Recently, for example, various heterostructure composites, such as Ag/g-C_3_N_4_ [29,30,31], Au/g-C_3_N_4_ [31,32,33], graphene oxide/g-C_3_N_4_ [34,35,36,37], TiO_2_/g-C_3_N_4_ [38,39,40,41,42], Ag/TiO_2_/g-C_3_N_4_ [43,44,45], TiO_2_/Cu/g-C_3_N_4_ [46], TiO_2_/ZrO_2_/g-C_3_N_4_ [47], and Bi_2_WO_6_/g-C_3_N_4_/TiO_2_ [48] have been successfully prepared, to optimize the optical properties of g-C_3_N_4_ and significantly improve its overall photocatalytic activity.

g-C_3_N_4_ and g-C_3_N_4_-containing heterostructure composites have also become important materials in the field of textiles and can be beneficially used for the degradation of pollutants in textile wastewater or for the chemical modification of textile fibers, to create different functionalities [11]. Natural and synthetic textile fibers are an ideal material for the fabrication of textile-based photocatalytic platforms, because they have advantages over other solid substrates, such as flexibility, lightness, porosity, absorptivity, and wearability. However, while the photocatalytic degradation of different dyes through the presence of g-C_3_N_4_ alone or its heterostructure composites has become a widely used sustainable strategy for the purification of dye wastewater [11], the functionalization of textile fibers with g-C_3_N_4_-containing materials remains challenging and, therefore, a topic of research [27]. There are 65 publications dealing with the use of g-C_3_N_4_ and g-C_3_N_4_ heterostructures for textile applications (Source: Web of Science, advanced search query preview: “graphitic carbon nitride or g-C_3_N_4_” and “textile or fabric” in abstracts, assessed on 28 December 2022), but most of these studies deal with the removal of textile dyes from wastewater. Therefore, it is of great importance to investigate the advantages of g-C_3_N_4_ and g-C_3_N_4_-containing heterostructure composites as promising “green” materials for textile functionalization.

In our previous review paper [27], we presented g-C_3_N_4_ as a new sustainable photocatalyst for textile functionalization, focusing on the textile substrates used, the application methods, and the developed functionalities, such as photocatalytic self-cleaning, antibacterial, and flame-retardant properties, as well as the creation of a textile catalytic platform for water disinfection, removal of various organic pollutants from water, and selective organic matter transformations. To provide additional valuable information on the recent advances in surface and bulk modification of textile fibers by g-C_3_N_4_-containing nanomaterials, this review article focuses on the application of heterostructure nanocomposites of g-C_3_N_4_ with Ag and TiO_2_ nanoparticles (NPs), as the most popular and widely used nanomaterials for surface and bulk chemical modification of textiles [49]. In the literature, both binary and ternary heterostructure composites, including Ag/g-C_3_N_4_, TiO_2_/g-C_3_N_4_, and Ag/TiO_2_/g-C_3_N_4_ have been considered promising functional nanomaterials, because the synergistic effect of the components in the heterostructures leads to the enhanced photocatalytic performance of the composites compared with the single-component materials. In this review, the processes for the synthesis of Ag/g-C_3_N_4_, TiO_2_/g-C_3_N_4_, and Ag/TiO_2_/g-C_3_N_4_ nanocomposites are explained, while their potential photocatalytic mechanisms of action and the developed functionalities on textile fibers for photochemical activation of organic synthesis, energy generation, and the removal of various organic pollutants, as well as future prospects, are highlighted.

## 2. Ag/g-C_3_N_4_ Nanocomposites

### 2.1. Preparation and Photocatalytic Mechanism of Ag/g-C_3_N_4_ Nanocomposites

As a superior multifunctional nanomaterial, Ag NPs are attractive candidates for surface loading or doping to develop noble metal/semiconductor heterostructures, also referred to as Ag/g-C_3_N_4_ [50,51]. The Ag/g-C_3_N_4_ nanocomposites exhibit not only enhanced visible light photocatalytic performance, but also improved antimicrobial performance due to the excellent antimicrobial activity of Ag against a wide range of Gram-negative and Gram-positive bacteria, viruses, fungi, molds, yeasts, and algae [52]. Ag/g-C_3_N_4_ nanocomposites have already been used for the degradation of environmental pollutants, such as in the decolourization of different dyes [30,53,54,55,56,57,58] and the degradation of organic solvents [53,59,60,61] and antibiotics [62,63,64,65]. They have also been used in hydrogen generation [66]; in microbial disinfection [57,67,68]; and as chemical sensors to detect drugs [69,70], biothiols [71], plant pigments [72], and pesticides [61]. In another case, Ag/g-C_3_N_4_ was used to obtain composites with multiple colors [73].

**Figure 2:**
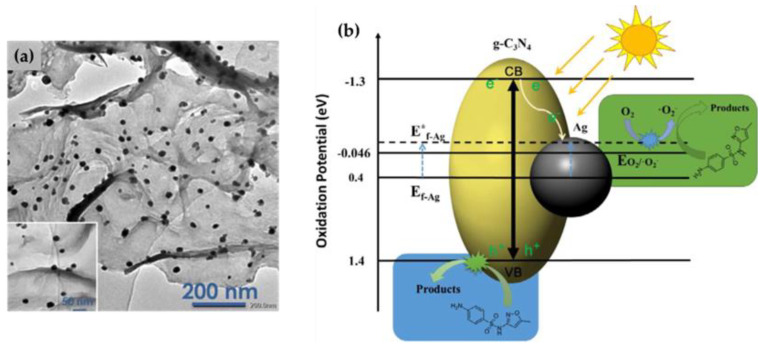
TEM image of Ag/g-C_3_N_4_ nanocomposite (**a**), photocatalytic mechanism of the Ag/g-C_3_N_4_ heterostructure (**b**). Reprinted with permission from [63]. Copyright 2018, Elsevier.

Two different approaches have been used to prepare Ag/g-C_3_N_4_ nanocomposites, namely one-step and two-step processes, the latter of which is more commonly used. In the two-step process [30,53,54,55,56,57,59,61,63,65,67,69,70,71,72,73], g-C_3_N_4_ is first synthesized from a suitable precursor in the form of bulk material, g-C_3_N_4_ nanosheets, or g-C_3_N_4_ quantum dots. Then, g-C_3_N_4_ is dispersed in the water medium and mixed with AgNO_3_, which serves as a precursor for Ag NPs. Subsequently, Ag NPs are synthesized in the presence of g-C_3_N_4_ using various reducing agents, such as NaBH_4_ [30,59,61,65], hydrazine hydrate [70], sodium citrate [72], plant extracts [54], and UV light [53,55,63,67,73]. In this case, g-C_3_N_4_ serves as a platform for the synthesis of Ag NPs, and its surface is decorated with Ag^0^ (Figure 2a). On the other hand, in the one-step process [56,57,62,74], urea or a mixture of melamine and cyanuric acid are used as g-C_3_N_4_ precursors and mixed with AgNO_3_ in a suitable medium, and the simultaneous synthesis of Ag NPs/g-C_3_N_4_ is carried out under appropriate conditions. This synthesis procedure enables the preparation of Ag-doped g-C_3_N_4_.

The proposed mechanism behind the photocatalytic activity of the Ag/g-C_3_N_4_ nanocomposite is shown in Figure 2b [53,54,56,57,59,63,75,76]. It is believed that the photocatalytic efficiency of the noble metal/semiconductor heterostructure composite is significantly enhanced by the presence of Ag^0^, which acts as a current collector and plasmonic absorber [77].

Ag/g-C_3_N_4_ nanocomposite is a visible-light photocatalyst. According to the literature [23], the band gap energy of g-C_3_N_4_ is 2.7 eV, with the potentials of the valence band (VB) and conduction band (CB) being 1.4 eV and −1.3 eV, respectively. Irradiation with energy higher than the band gap energy of g-C_3_N_4_ results in the excitation of electrons (e^−^) from VB to CB, leaving holes (h^+^) in VB. The photogenerated e^−^ in CB of g-C_3_N_4_ can be easily transferred to the Ag NPs because the Fermi level of Ag is less negative compared with the CB of g-C_3_N_4_. This creates a Schottky barrier that maximizes photoinduced charge carrier separation and prevents recombination of the e^−^-h^+^ pair. The injected e^−^ accumulates on Ag and can easily enter the reduction reactions on the surface, such as the reduction of O_2_ to superoxide radicals ($•O_{2}^{-}$) [63] or the reduction of H^+^ to H_2_ in hydrogen production by water splitting [76]. At the same time, visible light leads to the excitation of e^−^ in the Ag surface layer, resulting in surface plasmon resonance (SPR). The generation of SPR can greatly enhance the photoactivity of the composite through the mechanism of plasmon resonance energy transfer [77], because the intense near-electric field induced by SPR improves the efficiency of charge carrier separation [33] and increases the rate of charge carrier formation in g-C_3_N_4_ [44]. On the other hand, h^+^ in VB can directly oxidize different pollutants [57,63], but h^+^ cannot oxidize ^−^OH to give •OH radicals, since the VB edge potential of g-C_3_N_4_ is less positive than the standard redox potential of ^−^OH/•OH (+1.99 eV) [53,57,63,75]. This means that •OH radicals cannot be directly generated in the photochemical process of g-C_3_N_4_.

### 2.2. Ag/g-C_3_N_4_ Nanocomposites for Textile Application

Chemically modified textile substrates with Ag/g-C_3_N_4_ nanocomposites have been advantageously used in the photocatalysis of various organic reactions [78,79] and textile-based triboelectric nanogenerators [80]. While the first application involves photochemical activation of organic synthesis without additional reagents, thus providing a more environmentally friendly route for organic chemical conversion, the second application represents an emerging textile-based energy-harvesting device, as a potential power source for wearable electronics.

**Figure 3:**
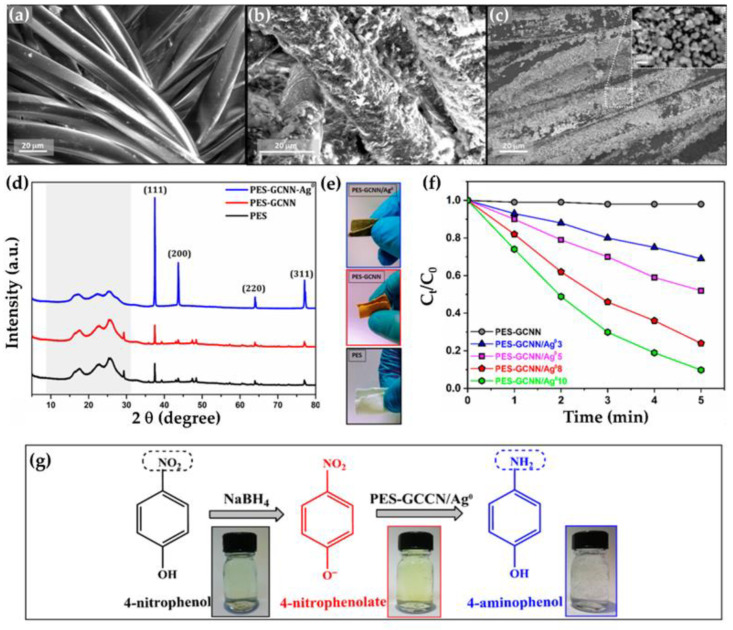
SEM images of uncoated PES fabric (**a**), PES coated with g-C_3_N_4_ (PES-GCNN sample) (**b**), and PES coated with Ag/g-C_3_N_4_ nanocomposite containing 10 wt% of Ag^0^ (PES-GCNN-Ag^0^ 10) (**c**); XRD patterns of PES, PES-GCNN, and PES-GCNN-Ag^0^ 10 samples (**d**); photos of PES, PES-GCNN, and PES-GCNN-Ag^0^ 10 samples (**e**); time-dependent 4-NP conversion over PES-GCNN-Ag^0^ samples containing 3, 5, 8, and 10 wt% of Ag^0^ (**f**); reaction scheme and photographs representing reduction of 4-NP to 4-AP by NaBH_4_ catalyzed by PES-GCNN-Ag^0^ (**g**). Reprinted with permission from [79]. Copyright 2021, MDPI.

New functionality was imparted to a polyester (PES) fabric by dip-coating with g-C_3_N_4_ nanosheets under ultrasonic treatment, followed by in situ synthesis of Ag NPs in an aqueous solution of AgNO_3_ of different concentrations (3–10 wt%), using NaBH_4_ as a reducing agent [79]. From the SEM images, it is evident that the micro smooth morphology of the PES fibers (Figure 3a) was completely changed by the application of the g-C_3_N_4_ nanosheets (Figure 3b), as well as the Ag/g-C_3_N_4_ nanocomposite (Figure 3c). The latter became microrough, with clearly visible deposited and uniformly distributed spherical Ag^0^ particles with an average size of 13.3 nm. The crystalline phase of the uncoated and coated PES samples was determined by X-ray diffraction (Figure 3d). Since the characteristic diffraction peaks of g-C_3_N_4_ at 2θ~27.5° and 13.1° could not be detected in the XRD pattern of the PES sample, the presence of Ag^0^ showed four peaks at 2θ = 37.47°, 43.69°, 63.97°, and 77.02°, corresponding to the cubic Ag^0^ planes (111), (200), (220), and (311), respectively. It is also evident that the application of g-C_3_N_4_ nanosheets changed the white color of the PES fabric to brown, but the in situ synthesis of Ag NPs to grey (Figure 3e).

PES coated with g-C_3_N_4_ and Ag/g-C_3_N_4_ nanocomposites, containing different amounts of Ag^0^ from 3 to 10 wt%, was used as a sustainable chemical catalyst for the hydrogenation of 4-nitrophenol, one of the most toxic organic pollutants in industrial wastewater, into the valuable compound 4-aminophenol, using NaBH_4_ as the hydride source [79]. Since a one-component g-C_3_N_4_ coating on PES fabric does not act as a catalyst for the conversion of 4-nitrophenol into 4-aminophenol, the presence of 3 wt% Ag^0^ in the Ag/g-C_3_N_4_ nanocomposite resulted in a 30% conversion of 4-nitrophenol into 4-aminophenol after a reaction time of 5 min, and this increased to 90% conversion when the Ag loading was increased to 10 wt%, with an apparent rate constant of 0.462 min^−1^, which is more than six-times higher than that of 3 wt% Ag^0^. (Figure 3f). This indicates that Ag NPs facilitated the electron transfer from ${\mathrm{BH}}_{4}^{-}$ to 4-nitrophenolate, thus lowering the barrier of activation energy for the reduction of 4-nitrophenol to 4-aminophenol (Figure 3g). The high recyclability and stability of the catalyst was evidenced by the fact that the catalytic performance of the catalyst was still nearly 90% after 10 cycles. A comparison with some other Ag-based catalysts from the literature clearly showed that the conversion of 4-nitrophenol over Ag/g-C_3_N_4_ coated PES exhibited enhanced the catalytic activity and recyclability [79].

**Figure 4:**
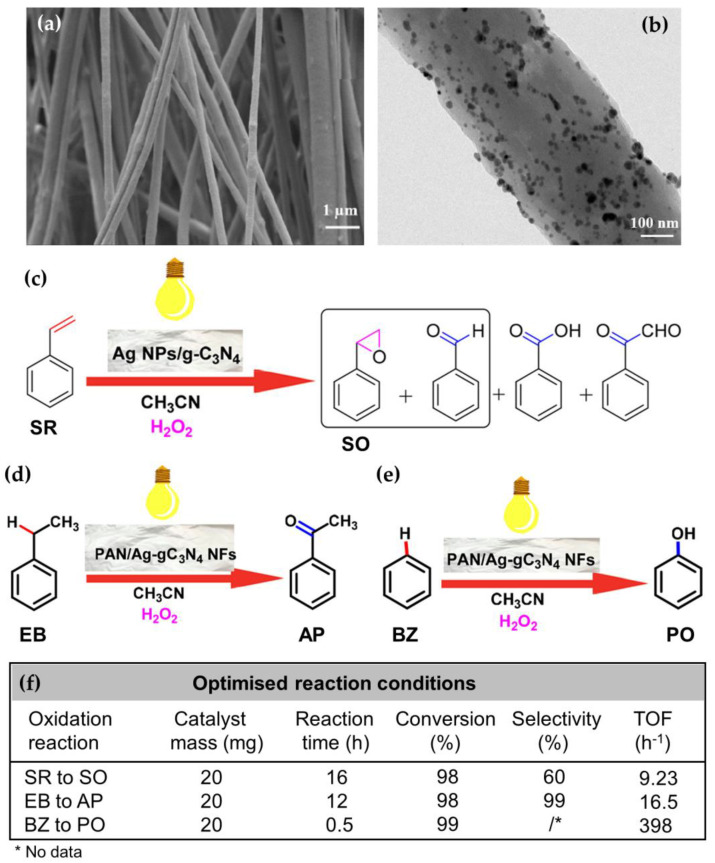
SEM (**a**) and TEM (**b**) images of PAN NFs with the embedded Ag NPs/g-C_3_N_4_; schematic presentation of oxidation of styrene (SR) to styrene oxide (SO) (**c**), selective oxidation of ethylbenzene (EB) to acetophenone (AP) (**d**), and selective oxidation of benzene (BZ) to phenol (PN) (**e**) by PAN NFs, with the embedded Ag NPs/g-C_3_N_4_ as a catalyst under visible light irradiation, and optimized reaction conditions with the turnover frequency (TOF) values (**f**). Reprinted with permission from [78]. Copyright 2020, ACS.

Ag NP-decorated g-C_3_N_4_ was also used in the bulk modification of polyacrylonitrile nanofibers (PAN NFs) for selective oxidation of styrene, benzylic methylene groups, and benzene into the desired products under visible light irradiation and milder reaction conditions [78]. For this purpose, Ag NPs/g-C_3_N_4_ composite and 10 wt% PAN were dispersed in an organic solvent under sonification, to produce a homogeneous polymer solution, which was then electrospun to produce PAN NFs with the embedded Ag NPs/g-C_3_N_4_ (Figure 4a,b). TEM micrography showed small dark particles and bulges on the PAN NFs, indicating that the Ag NPs/g-C_3_N_4_ was well dispersed on the PAN surface or embedded in the PAN matrix, without agglomerating (Figure 4b). The as-prepared PAN/Ag NPs/g-C_3_N_4_ NFs exhibited a highly porous nature with excellent absorption performance. To optimize the photooxidation reactions, the influence of different parameters, such as the amount of PAN/Ag NPs/g-C_3_N_4_ NFs, organic solvents, reaction time, the presence or absence of visible light (domestic bulb (40 w)), and H_2_O_2_ as oxidant, on the photocatalytic performance was investigated. It was found that the oxidation selectivity and conversion increased with the increase in catalyst concentration and reaction time in the organic solvent acetonitrile, as well as in the presence of light and H_2_O_2_. In this case, a 98% conversion of styrene and 60% selectivity toward styrene oxide (Figure 4c), 98% conversion of ethylbenzene and 99% selectivity toward acetophenone (Figure 4d), as well as an excellent 99% conversion of benzene into the desired phenol, was obtained using H_2_O_2_ as oxidant (Figure 4e) under optimized reaction conditions (Figure 4f). A recyclability study of the PAN/Ag NPs/g-C_3_N_4_ NFs for styrene oxidation showed that the catalyst exhibited high reusability activity with a product yield of more than 85% in a repeat test of five runs.

**Figure 5:**
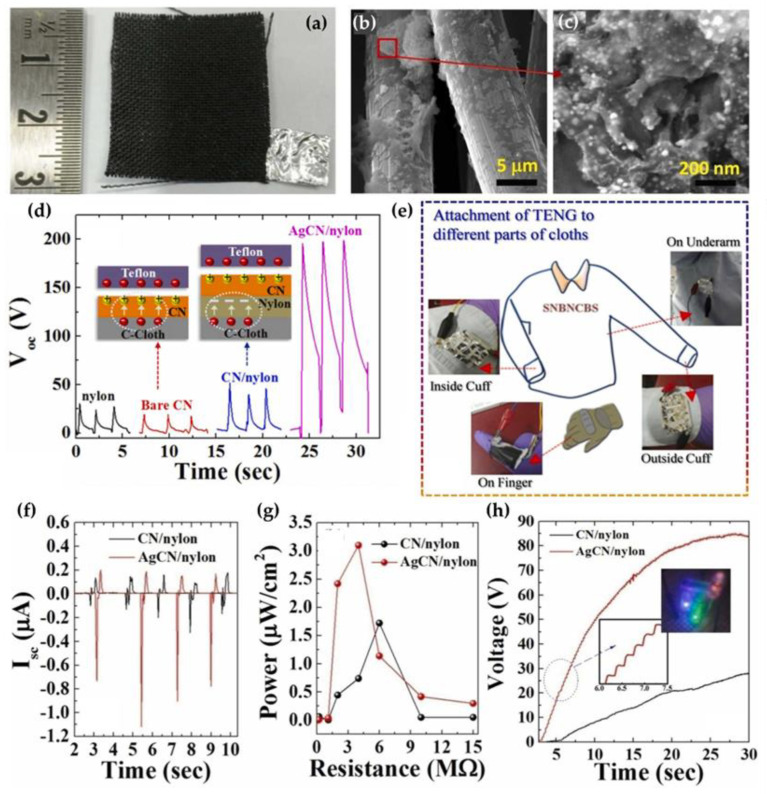
Photograph of an Ag/g-C_3_N_4_/nylon coated C-cloth with Al electrode (**a**); SEM images of Ag/g-C_3_N_4_ decorated on nylon coated C-cloth under lower and higher magnification (**b**,**c**); open circuit voltage generation from TENG operation for different layers with Teflon as a counter triboelectric material under a mechanical impact (**d**); attachment of TENG to different parts of clothes (**e**); short circuit current (**f**), output power density (**g**), and capacitor charging voltage (**h**) for g-C_3_N_4_/nylon and Ag/g-C_3_N_4_/nylon based T-TENGs (insets show a closer view of voltage profile and the glowing of LEDs using the charged capacitor). Reprinted with permission from [80]. Copyright 2022, Elsevier.

Recently, a textile-based triboelectric nanogenerator (T-TENG) for mechanical energy harvesting was fabricated by depositing an active layer of g-C_3_N_4_ nanosheets decorated with Ag NPs on a nylon-coated conductive carbon fabric as a textile backbone (Figure 5a–c), using Teflon or polypropylene as a counter triboelectric material [80]. To establish electrical contact, aluminum adhesive tapes were attached on one of the surfaces of these layers, with an extension for electrical contacts. To produce a voltage, the fabric based samples and Teflon were put in repetitive contact-separation mode through mechanical agitation. When the two materials are in contact, charge transfer occurs between the two surfaces, and when the two materials are separated, a current flows through the external circuit, to balance the potential on the two surfaces. Such a configuration of an Ag/g-C_3_N_4_/nylon bi-layer T-TENG generates an open circuit voltage of ~200 V, which is more than 10 times higher compared with a bare g-C_3_N_4_ nanosheet layer (19 V) and almost four-times higher compared to a g-C_3_N_4_/nylon bi-layer (52 V) (Figure 5d). The thermal stability, as well as the conversion efficiency, at an elevated temperature of up to ~65 °C make it a potential candidate for integration into textile-based wearable electronic devices (Figure 5e). The synergistic effect of interfacial charge trapping, the increased surface area and the increased surface charge density in the Ag/g-C_3_N_4_/nylon bi-layer system result in the development of a maximum short circuit current of ~1.1 μA, which is about three-times higher than that of g-C_3_N_4_/nylon (Figure 5f) and delivery of a maximum output power of ~3.1 μW/cm^2^, which is higher than that obtained from g-C_3_N_4_/nylon (Figure 5g). When a Ag/g-C_3_N_4_/nylon bi-layer T-TENG was examined upon charging a commercial capacitor (0.26 μF) using a bridge rectifier, the Ag/g-C_3_N_4_/nylon device was able to charge the capacitor to ~85 V within 30 s, which is more than 2.7 times higher than the result of a g-C_3_N_4_/nylon device (Figure 5h). In addition, several commercially available LEDs could be driven by the capacitors, which were charged by the impact of the textile-based nanogenerator (insert in Figure 5h). The excellent power generation capability of this fabric coated with an Ag/g-C_3_N_4_/nylon bi-layer indicates its potential applicability for wearable and flexible nanogenerators.

## 3. TiO_2_/g-C_3_N_4_ Heterojunctions

### 3.1. Preparation and Photocatalytic Mechanism of TiO_2_/g-C_3_N_4_ Nanocomposites

The design and construction of TiO_2_/g-C_3_N_4_ hybrid photocatalysts have attracted much attention, as they have been recognized as an effective material for various environmental and energy applications [81,82,83,84,85,86,87,88,89,90,91,92,93,94,95,96,97,98]. It should be noted that TiO_2_ in the TiO_2_/g-C_3_N_4_ heterojunction is a wide-band-gap semiconductor that responds to UV light (Eg = 3.2 eV; λ ≤ 387.5 nm) [99,100], while g-C_3_N_4_ responds to visible light. It is believed that the formation of a synergistic TiO_2_/g-C_3_N_4_ heterojunction can significantly reduce the recombination of photogenerated electron–hole pairs and increase the photocatalytic activity of TiO_2_ in visible light, which is beneficial for both photocatalysts [92,101]. Moreover, the TiO_2_/g-C_3_N_4_ heterojunction is expected to simultaneously utilize UV and visible light, thus exhibiting excellent photocatalytic performance under UV and visible light irradiation [82,92].

In the preparation of a TiO_2_/g-C_3_N_4_ heterojunction, two-step processes have generally been applied in two different ways [82,83,84,85,86,87,88,91,92,93,94,95,96,97,98]. The first approach involves the prior synthesis of both TiO_2_ and g-C_3_N_4_ from the corresponding precursors and subsequent mixing [87,88,92,95,96,97,98]. In the other approach, in situ synthesis of TiO_2_ was performed in the presence of presynthesized g-C_3_N_4_ [82,83,84,85,86,93,94] or conversely, the in situ synthesis of g-C_3_N_4_ was performed in the presence of presynthesized TiO_2_ [91]. While urea [84,85,88,91,92,95], melamine [82,86,93,94,96,97], dicyandiamide [87,98], or a combination of urea and melamine [83] have been used as precursors for g-C_3_N_4_ synthesis, titanium(IV) butoxide [85,92,93,95,96], titanium(IV) isopropoxide [84], titanium tetrachloride [82,83,86], titanium(IV) bis-(ammonium lactato) dihydroxide [94], and titanyl sulphate [97] are widely used as TiO_2_ precursors. For TiO_2_ synthesis, the hydrothermal or solvothermal assisted sol-gel process under acidic or alkaline conditions is mostly used [82,84,86,87,92,93,96]. In addition to the two-step processes, a one-step hydrothermal process using melamine as g-C_3_N_4_ precursor and titanium(IV) isopropoxide as TiO_2_ precursor and cyanuric acid as catalyst has also been reported [90]. All the above processes are completed by drying and calcining the nanocomposites under suitable conditions, to obtain the desired morphology.

**Figure 6:**
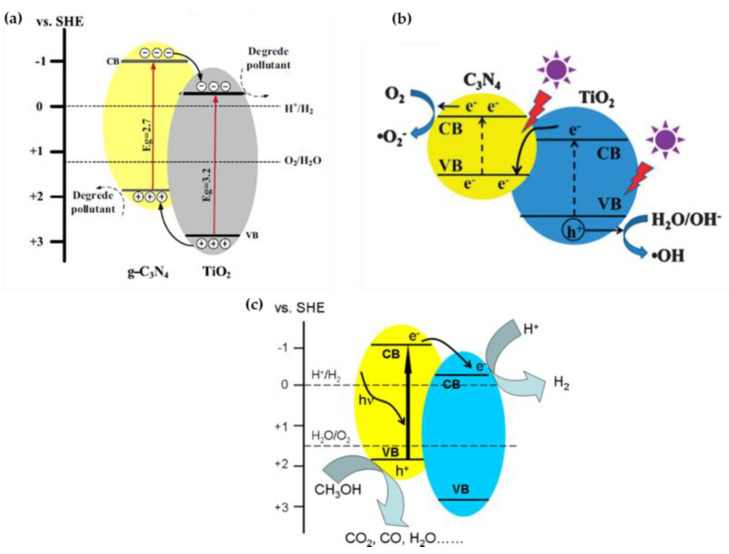
The photocatalytic mechanism of the TiO_2_/g-C_3_N_4_ heterojunction: Type II (**a**) (reprinted with permission from [82]; Copyright 2012, Elsevier), and Z-scheme (**b**) (reprinted with permission from [91]; Copyright 2013, RSC Publishing) under UV/visible light irradiation; electron transfer pathway under exclusive visible light irradiation (**c**). Reprinted with permission from [81]; Copyright 2011, Elsevier.

Two mechanisms have been proposed for the photocatalytic activity of TiO_2_/g-C_3_N_4_ nanocomposites, including the Type-II heterojunction (Figure 6a) [82,87,92,93,94,96,97,98,102] and the direct Z-scheme (Figure 6b) [90,91,95]. In both mechanisms, it is assumed that when g-C_3_N_4_ and TiO_2_ are excited in the heterojunction by incident UV/visible light of sufficient energy, the photoinduced electrons are transferred from VB to CB, leaving holes in VB. According to the Type-II heterojunction mechanism, the photogenerated electrons can be easily transferred from CB of g-C_3_N_4_ to CB of TiO_2_ because the CB edge potential of g-C_3_N_4_ (−1.3 eV) is more negative than that of TiO_2_ (−0.29 eV). At the same time, the photogenerated holes can be transferred from VB of TiO_2_ to VB of g-C_3_N_4_ because the VB edge potential of TiO_2_ (2.91 eV) is more positive than that of g-C_3_N_4_ (1.4 eV). In this case, photoinduced electrons accumulate in the CB of TiO_2_ for the reduction reaction and photoinduced holes accumulate in the VB of g-C_3_N_4_ for the oxidation reaction, which efficiently separates the photogenerated electron–hole pairs and suppresses their recombination [102,103,104]. However, despite the enhanced photocatalytic efficiency of the as-constructed TiO_2_/g-C_3_N_4_ nanocomposite, a drawback of the Type-II heterojunction mechanism is attributed to the impairment of redox capability, since the reduction reaction proceeds on TiO_2_ with a lower reduction potential compared with g-C_3_N_4_, and the oxidation reaction proceeds on g-C_3_N_4_ with a lower oxidation potential compared with TiO_2_ [102,103]. Since the holes in the VB of g-C_3_N_4_ cannot directly generate OH radicals in the oxidation reaction, this significantly reduces the photocatalytic efficiency of the nanocomposite.

In contrast to the Type-II heterojunction mechanism, the direct Z-scheme photocatalysis system assumes a significantly different charge carrier transfer pathway in the TiO_2_/g-C_3_N_4_ nanocomposite, although it has the same band structure configuration (Figure 6b) [102]. Indeed, the direct Z-scheme dictates that the existence of an internal electric field, the extra potential barrier, and the Coulomb repulsion hinder the transfer of the photogenerated electrons from the CB of g-C_3_N_4_ to the CB of TiO_2_ and the photogenerated holes from the VB of TiO_2_ to the VB of g-C_3_N_4_, as well as promoting the recombination between the photogenerated electrons in the CB of TiO_2_ and the photogenerated holes in the VB of g-C_3_N_4_ with a lower redox ability [102,103]. In this case, the electrons and holes are spatially separated on g-C_3_N_4_ with the higher reduction potential and TiO_2_ with a higher oxidation potential, respectively. The electrons in the CB of g-C_3_N_4_ are trapped by O_2_ on the nanocomposite surface to form reactive $•O_{2}^{-}$, since the CB edge potential of g-C_3_N_4_ (−1.3 eV) is more negative than the redox potential of O_2_/$•O_{2}^{-}$ (−0.33 eV and −0.046 eV), while the holes in VB of TiO_2_ react with absorbed water to generate reactive •OH radicals [91] because the VB edge potential of TiO_2_ (2.91 eV) is more positive than the redox potential of ^−^OH/•OH (+1.99 eV). Compared with the Type-II heterojunction, the direct Z-scheme has a much stronger redox capability to drive photocatalytic reactions [102], which could explain the superior photocatalytic efficiency of TiO_2_/g-C_3_N_4_ nanocomposite in the various reduction and oxidation reactions. The presence of the direct Z-scheme instead of the Type-II heterojunction was also confirmed by experiments involving the trapping of •OH and $•O_{2}^{-}\;$radicals [103].

On the other hand, when irradiated with visible light, when only g-C_3_N_4_ can be excited because the energy of the incident light is too low to excite TiO_2_, the reduction reaction on the TiO_2_ surface can be indirectly induced via electron transfer from the CB of g-C_3_N_4_ to the CB of TiO_2_, leading to effective separation of photogenerated electron–hole pairs and an enhanced photocatalytic activity (Figure 6c) [81,84,96].

### 3.2. TiO_2_/g-C_3_N_4_ Nanocomposites for Textile Application

**Figure 7:**
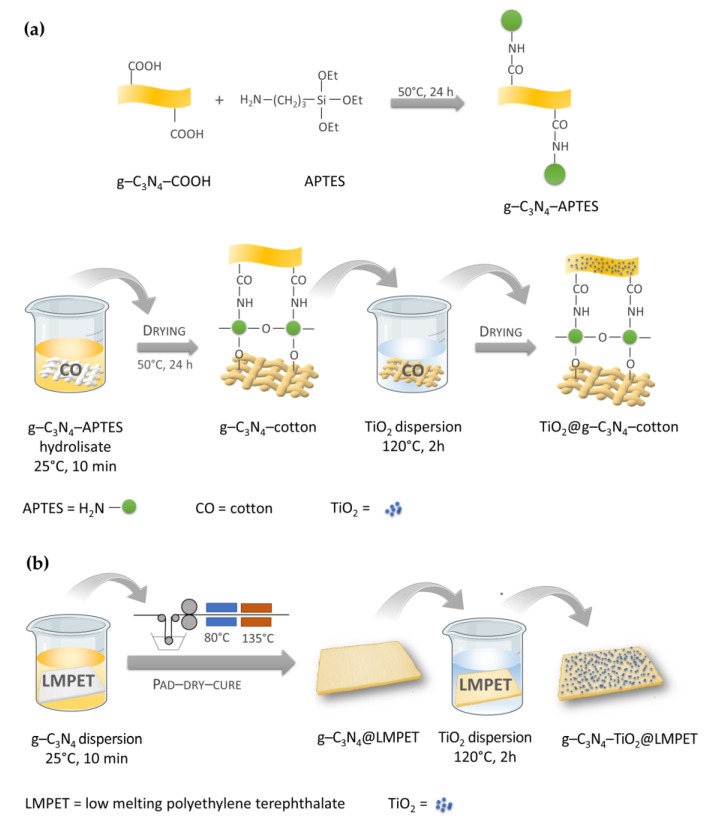
Schematic presentation of the preparation of the TiO_2_@g-C_3_N_4_–cotton (**a**) and the g-C_3_N_4_-TiO_2_@LMPET (**b**) photocatalysts (prepared according to refs. [106,107].

TiO_2_/g-C_3_N_4_ nanocomposites were applied to cotton and polyester substrates to develop textile-based photocatalysts for effective purification of emerging liquid, gaseous pollutants, and bacteria [39,105,106,107]. To this end, TiO_2_/g-C_3_N_4_–cotton and TiO_2_/g-C_3_N_4_–polyester composites were constructed and used as solar-driven photocatalysts for the degradation of the antibiotic sulphaquinoxaline (SQX) and the pesticide thiamethoxam (Figure 7) [106,107]. The preparation of a TiO_2_@g-C_3_N_4_–cotton photocatalyst included binding of the coupling agent (3-Aminopropyl)triethoxysilane (APTES) to the carboxyl-modified g-C_3_N_4_, to create reactive silanol groups on the g-C_3_N_4_ surface. Afterwards, a cotton fabric sample was immersed in the g-C_3_N_4_–APTES hydrolysate, followed by squeezing on a two-roll padder and drying at 130 °C, to chemically bind g-C_3_N_4_–APTES to the cotton surface. To produce the TiO_2_@g-C_3_N_4_–cotton, a g-C_3_N_4_–APTES-cotton sample was immersed in the TiO_2_ dispersion and maintained at 120 °C for 2 h for hydrothermal reaction, to achieve the deposition of TiO_2_ on the g-C_3_N_4_–APTES-cotton surface (Figure 7a). In the preparation of the g-C_3_N_4_-TiO_2_@LMPET photocatalyst, low-melting non-woven polyester (LMPET) was immersed in the g-C_3_N_4_ dispersion, followed by squeezing and drying at 80 °C, and heat treated at 135 °C, to melt the LMPET sheath to strongly stick g-C_3_N_4_. The as-prepared g-C_3_N_4_@LMPET was immersed in the TiO_2_ dispersion and maintained at 120 °C for 2 h for hydrothermal reaction, to achieve the deposition of TiO_2_ on the g-C_3_N_4_@LMPET surface (Figure 7b).

**Figure 8:**
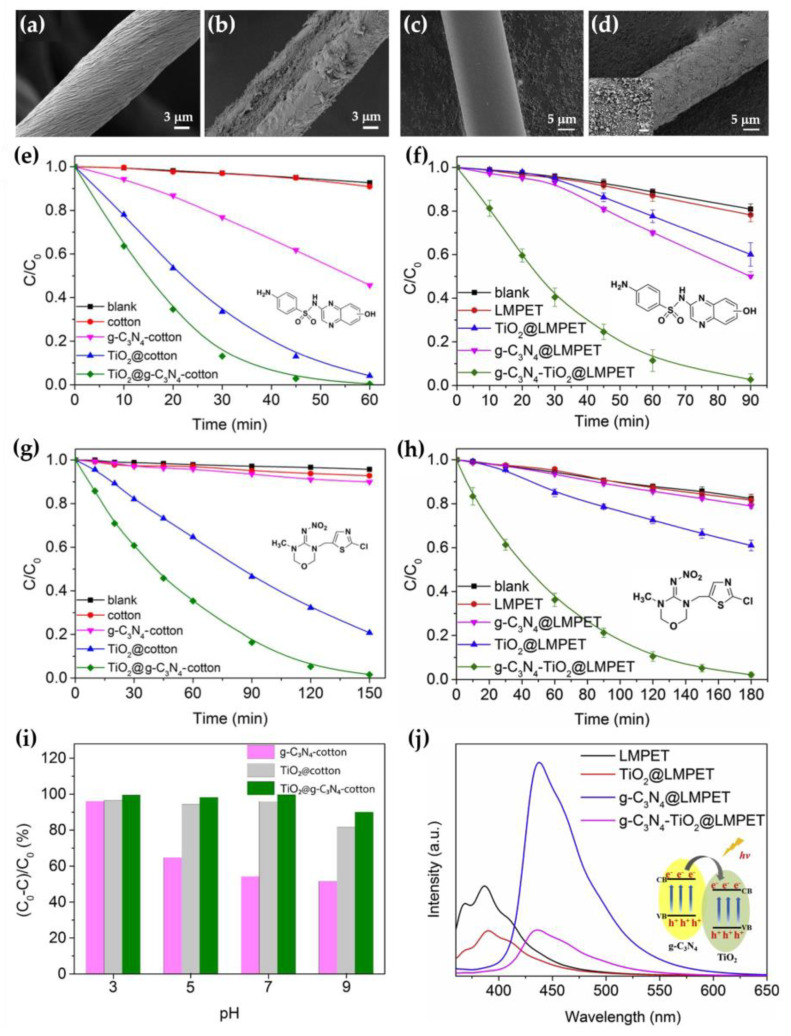
SEM images of cotton (**a**), TiO_2_@g-C_3_N_4_–cotton (**b**), LMPET (**c**), and g-C_3_N_4_-TiO_2_@LMPET (**d**) samples; photocatalytic degradation of SQX by cotton-based photocatalysts (**e**) and LMPET-based photocatalysts (**f**) under solar irradiation, [SQX] = 2 × 10^−5^ mol/L, pH 7; photocatalytic degradation of thiamethoxam by cotton-based photocatalysts (**g**), and LMPET-based photocatalysts (**h**) under solar irradiation, [thiamethoxam] = 2 × 10^−5^ mol/L, pH 7; photocatalytic degradation rate of SQX by cotton-based photocatalysts at different pH under solar irradiation for 60 min, [SQX] = 2 × 10^−5^ mol/L (**i**); photoluminescence spectra of LMPET and LMPET-based photocatalysts (**j**). (**a**,**b**,**e**,**g**,**i**) Reprinted with permission from [107]; Copyright 2021, Elsevier; (**c**,**d**,**f**,**h**,**j**) Reprinted with permission from [106], Copyright 2019, Elsevier.

The incorporation of the TiO_2_/g-C_3_N_4_ nanocomposite into the cotton and LMPET significantly changed the morphology of the fibers, resulting in an increased surface roughness of the cotton (Figure 8a–d) [107] as well as LMPET [106]. Photocatalytic activity was studied in the degradation of the antibiotic sulphaquinoxaline (SQX) (Figure 8e,f) and the pesticide thiamethoxam (Figure 8g,h) under sunlight irradiation. It was found that both the TiO_2_@g-C_3_N_4_-cotton and g-C_3_N_4_-TiO_2_@LMPET samples showed excellent photocatalytic performance, resulting in an almost 100% degradation of SQX after 60 and 90 min by the TiO_2_@g-C_3_N_4_-cotton and g-C_3_N_4_-TiO_2_@LMPET samples, respectively, and of thiamethoxam after 150 and 180 min by TiO_2_@g-C_3_N_4_-cotton and g-C_3_N_4_-TiO_2_@LMPET samples, respectively, at pH 7. The photocatalytic performance was sufficiently higher than that of the TiO_2_-cotton and TiO_2_@LMPET samples and that of the g-C_3_N_4_-cotton and g-C_3_N_4_@LMPET samples. The rate of SQX removal by TiO_2_/g-C_3_N_4_ was higher than that of thiamethoxam for both textile-based photocatalysts, because thiamethoxazine is a more difficult pollutant to degrade than SQX. The results also showed that the TiO_2_/g-C_3_N_4_ composite exhibited better photocatalytic activity in the decomposition of SQX under acidic and neutral conditions and gradually weakened under alkaline conditions (Figure 8i). Both TiO_2_@g-C_3_N_4_-cotton and g-C_3_N_4_-TiO_2_@LMPET photocatalysts maintained excellent catalyst recyclability and stability and could remove 97% SQX after 10 cycles.

The mechanism of the photocatalytic activity of the TiO_2_/g-C_3_N_4_ heterojunction and the behavior of the charge transfer at the interface were discussed based on the photoluminescence spectra (Figure 8j) [106]. The results showed that g-C_3_N_4_ exhibited a strong emission peak at about 450 nm, which decreased drastically in the case of the TiO_2_/g-C_3_N_4_ heterojunction. This phenomenon can be explained by the electron transfer from the CB of g-C_3_N_4_ to the CB of TiO_2_, which efficiently suppresses the recombination of photogenerated electron–hole pairs and improves the photocatalytic performance compared with the single-components TiO_2_ and g-C_3_N_4_, indicating a synergistic effect between TiO_2_ and g-C_3_N_4_ in the heterojunction.

**Figure 9:**
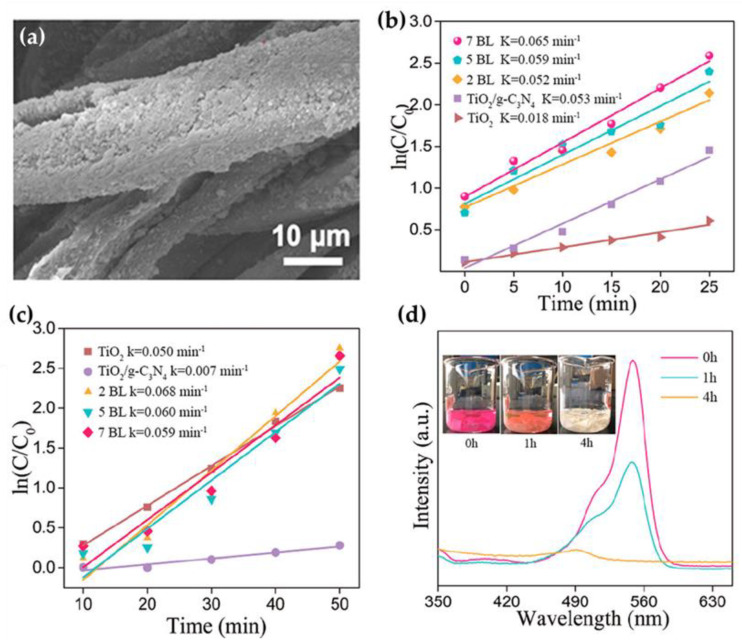
SEM images of the cotton modified with TiO_2_/g-C_3_N_4_ (**a**); kinetic degradation curves of RhB under visible-light irradiation (**b**), kinetic degradation curves of toluene under simulated sunlight irradiation (**c**); absorption spectra of RhB under real sunlight (**d**). Reprinted with permission from [105]. Copyright 2019, ACS Publications.

A textile-based photocatalyst was also prepared by constructing a TiO_2_/g-C_3_N_4_ coating on cotton fabric using a simple layer-by-layer (LBL) self-assembly strategy, in which the cotton fabric was alternately immersed in the cationic TiO_2_ solution and the anionic g-C_3_N_4_ solution to obtain two, five, and seven bilayers (BL) [105]. After each immersion, the sample was rinsed and dried. A TiO_2_/g-C_3_N_4_ powder composite was also prepared for comparison. The SEM analysis revealed that the self-assembly coating mainly covered the surface of the cotton fibers and significantly increased their roughness (Figure 9a). The mass of the coating increased with the number of BL. The photocatalytic performance of the TiO_2_/g-C_3_N_4_ coated cotton fabric was investigated through the degradation rate of RhB dye (Figure 9b) and toluene (Figure 9c) under visible-light irradiation. The higher the degradation rate constant, the higher the photocatalytic performance. From the results, it can be seen that the reaction rate constant, κ, for the degradation of Rhodamine B (RhB) dye gradually increased with the increasing number of BL, and the highest value was reached for the coating with 7 BL, which was much higher than that of TiO_2_/g-C_3_N_4_ and TiO_2_ powders (Figure 9b). This confirmed that the coupling of TiO_2_ with g-C_3_N_4_ is an efficient strategy to improve photocatalytic performance and highlights the importance of the cotton fabric as a support for the photodegradation reaction, since the fabric acts as an absorbent for pollution and drives the active species to rapidly absorb and degrade the pollutants. Similar results were obtained for the photodegradation of toluene, with coatings of 2 BL, 5 BL, and 7 BL showing significantly higher degradation compared with the TiO_2_/g-C_3_N_4_ powder (Figure 9c). The excellent performance of the 7 BL coated cotton fabric was also demonstrated by the degradation of RhB solution under sunlight, where not only was the RhB solution completely discolored within 4 h but also the coated fabric, indicating RhB degradation in the solution and on the fabric surface (Figure 9d).

**Figure 10:**
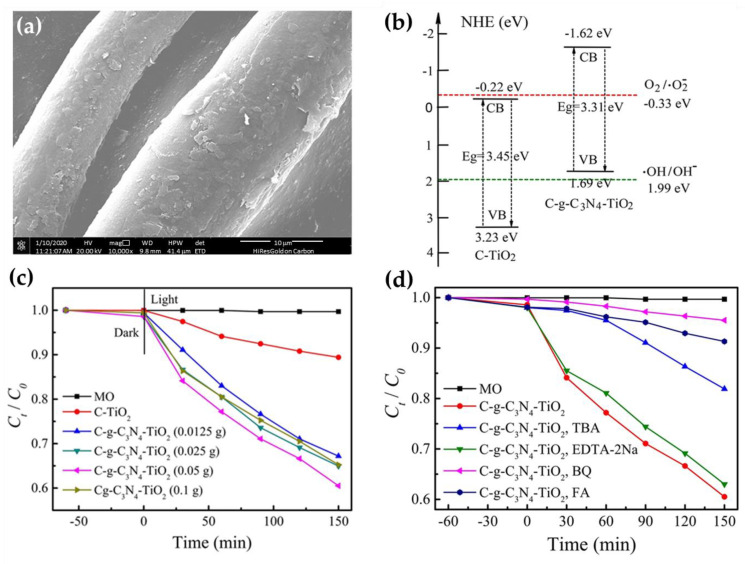
FESEM images of C–g-C_3_N_4_–TiO_2_ cotton fibers (**a**); schematic diagrams of energy band structure for C–TiO_2_ and C–g-C_3_N_4_–TiO_2_ cotton fibers (**b**); photodegradation of MO dye solution by the C–g-C_3_N_4_–TiO_2_ cotton fibers (**c**); trapping experiments for the photodegradation of MO dye solution by C–g-C_3_N_4_–TiO_2_ cotton fibers (**d**). Reprinted with permission from [39]. Copyright 2021, Springer Link.

To investigate the photocatalytic mechanism, a TiO_2_/g-C_3_N_4_ nanocomposite was chemically grafted onto cotton fibers in a hydrothermal process (Figure 10) [39]. In the preparation of cotton fabric loaded with TiO_2_/g-C_3_N_4_ in different mass ratios (C–g-C_3_N_4_–TiO_2_ samples), the in situ synthesis of TiO_2_ was performed in a solution of presynthesized g-C_3_N_4_ nanosheets in the presence of the swollen cotton fibers at 120 °C for 4 h. The results showed that the as-prepared C–g-C_3_N_4_–TiO_2_ samples exhibited fish-like lobes, with densely aggregated nanosized particles of irregular shape (Figure 10a). Chemical grafting of the g-C_3_N_4_–TiO_2_ heterojunction composite with cotton fibers resulted in an Eg of 3.31 eV, which was red-shifted compared with the Eg of C–TiO_2_ fibers of 3.45 eV (Figure 10b), indicating a higher light absorption efficiency. Both calculated Eg values were higher than those of TiO_2_ NPs (Eg = 3.2 eV) and g-C_3_N_4_ nanosheets (Eg = 2.82 eV), suggesting that cotton affected the energy band structure of the generated composite photocatalysts, which is difficult to explain.

The photocatalytic performance results of the C–g-C_3_N_4_–TiO_2_ and C–TiO_2_ samples showed that the C–TiO_2_ cotton fibers exhibited a poor photodegradation performance for methyl orange (MO) dye solution when irradiated with visible light, with only 10% MO decolorization after 150 min, while the photocatalytic performance of the C–g-C_3_N_4_–TiO_2_ cotton fibers was much higher (Figure 10c) [39]. The latter increased with the increase in the mass of the g-C_3_N_4_ nanosheets in the TiO_2_/g-C_3_N_4_ nanocomposite, from 0.025 to 0.05 g, and reached its maximum with a photocatalytic efficiency about four-times higher than that of the C–TiO_2_ cotton fibers. Further increasing the mass of the g-C_3_N_4_ nanosheets led to a decrease in the photocatalytic activity of the C–g-C_3_N_4_–TiO_2_ cotton fibers, indicating that the mass ratio between g-C_3_N_4_ and TiO_2_ in the heterojunction should be carefully selected.

The photocatalytic mechanism of action of the C–g-C_3_N_4_–TiO_2_ cotton fibers was determined through trapping experiments (Figure 10d) [39], in which photodegradation of the MO dye solution was performed under irradiation with visible light in the presence of four radical scavengers, i.e., 1,4-benzoquinone (BQ), furfuryl alcohol (FA), tertbutyl alcohol (TBA), and ethylenediaminetetraacetic acid disodium salt (EDTA-2Na) to scavenge $•O_{2}^{-}$, singlet oxygen (^1^$O_{2}$), •OH and h^+^, respectively. It was found that the photocatalytic activity of the C–g-C_3_N_4_–TiO_2_ cotton fibers decreased only slightly in the presence of EDTA-2Na, but a significant decrease in photocatalytic capacity was observed after the addition of BQ and FA. This indicates that $•O_{2}^{-}$, generated by the reduction reaction at the surface of the TiO_2_/g-C_3_N_4_ heterojunction, is the most important type of radical for the photocatalytic degradation of MO, followed by ^1^$O_{2}$, •OH, and h^+^. It was concluded that cotton fabric modified with g-C_3_N_4_–TiO_2_ can be repeatedly used to remove organic contaminants.

## 4. Ag/TiO_2_/g-C_3_N_4_ Heterostructure

### Preparation and Photocatalytic Mechanism of Ag/TiO_2_/g-C_3_N_4_ Nanocomposites

**Figure 11:**
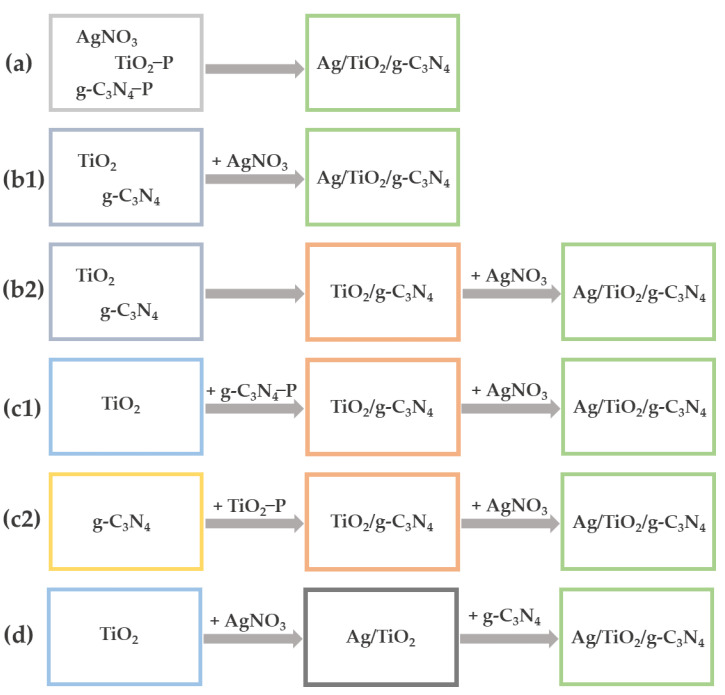
Schematic presentation of strategies for the synthesis of the Ag/TiO_2_/g-C_3_N_4_ nanocomposites: synthesis of Ag/TiO_2_/melamine from a mixture of Ag, TiO_2_ and g-C_3_N_4_ precursors, followed by calcination to produce Ag/TiO_2_/g-C_3_N_4_ (**a**); preparation of a mixture of TiO_2_ and g-C_3_N_4_, followed by synthesis of Ag NPs (**b1**); synthesis of TiO_2_/g-C_3_N_4_, followed by synthesis of Ag NPs (**b2**); synthesis of g-C_3_N_4_ in the presence of TiO_2_, followed by the synthesis of Ag NPs (**c1**); synthesis of TiO_2_ in the presence of g-C_3_N_4_, followed by the synthesis of Ag NPs (**c2**); synthesis of Ag NPs in the presence of TiO_2_, followed by a mixture with g-C_3_N_4_ (**d**). P = precursor.

The construction of ternary Ag/TiO_2_/g-C_3_N_4_ heterostructures represents a very promising strategy for achieving the enhanced photocatalytic performance of semiconductor nanocomposites under visible light irradiation [43,45,108,109,110,111,112,113,114,115,116,117,118,119,120,121,122,123,124,125,126]. Various approaches for the synthesis of Ag/TiO_2_/g-C_3_N_4_ nanocomposites have been reported in the literature. One of these proposes the preparation of a mixture of Ag, TiO_2_ and g-C_3_N_4_ precursors and the synthesis of an Ag/TiO_2_/melamine nanocomposite at 70 °C, followed by calcination of the nanocomposite at 550 °C, to produce g-C_3_N_4_ from melamine (Figure 11a) [126]. Another strategy is to mix previously synthesized TiO_2_ and g-C_3_N_4_, followed by the addition of an AgNO_3_ precursor and synthesis of Ag NPs in the presence of a g-C_3_N_4_ and TiO_2_ mixture or previously prepared TiO_2_/g-C_3_N_4_ nanocomposite (Figure 11b1,b2) [45,115,123,124]. A TiO_2_/g-C_3_N_4_ composite was also prepared by the synthesis of g-C_3_N_4_ from a suitable precursor in the presence of TiO_2_ or through the synthesis of TiO_2_ from a suitable precursor in the presence of g-C_3_N_4_, followed by the synthesis of Ag NPs from an AgNO_3_ precursor in a reduction reaction in the presence of TiO_2_/g-C_3_N_4_ nanocomposite (Figure 11c1,c2) [108,116,117]. It has also been reported that the Ag/TiO_2_/g-C_3_N_4_ nanocomposite was prepared by synthesis of Ag NPs from an AgNO_3_ precursor in the presence of TiO_2_ and subsequent mixing of the Ag/TiO_2_ nanocomposite with previously synthesized g-C_3_N_4_ (Figure 11d) [43,110,113,118,119,121,122].

**Figure 12:**
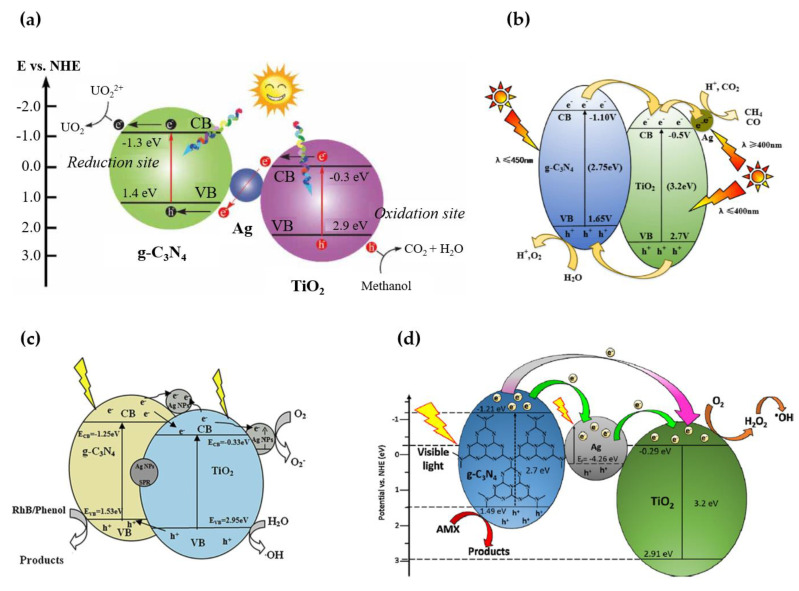
Direct Z-scheme of the photocatalytic mechanism of Ag/TiO_2_/g-C_3_N_4_ heterostructure (**a**) (Reprinted with permission from [45]; Copyright 2022, IWA Publishing); Type II heterojunction of Ag/TiO_2_/g-C_3_N_4_ accompanied by the Schottky barrier (**b**) (Reprinted with permission from [122]; Copyright 2017, Elsevier) and (**c**) (Reprinted with permission from [116], Copyright 2020, Elsevier); photocatalytic mechanism of Ag/TiO_2_/g-C_3_N_4_ under visible light (**d**) (Reprinted with permission from [120], Copyright 2015, Elsevier).

It should be emphasized that the photocatalytic mechanism of the ternary Ag/TiO_2_/g-C_3_N_4_ nanocomposite is very complex and not yet fully understood. Since it is influenced by nanocomposite construction, which is directly related to the synthesis route and the formation of tight interfacial connections between the components in the heterojunction, there are various schematic representations of the photocatalytic mechanisms of Ag/TiO_2_/g-C_3_N_4_ in the literature, as well as explanations of the charge carrier transfer. The most commonly proposed mechanisms are shown in Figure 12 [45,116,120,122].

It is proposed that the enhanced photocatalytic performance of Ag/TiO_2_/g-C_3_N_4_ is due to the effective Z-scheme mechanism established in the TiO_2_ and g-C_3_N_4_ heterojunction under UV- and visible-light irradiation, which is supported by the SPR of Ag facilitating charge transfer (Figure 12a) [45,108]. It has been suggested that Ag, as a conductive material, can directly act as a center to combine electrons on the surface of TiO_2_ with the holes on g-C_3_N_4_ and maintain this remarkable Z-scheme photocatalytic system [45].

Another possible photocatalytic mechanism of Ag/TiO_2_/g-C_3_N_4_ involves the Type-II mechanism of the TiO_2_/g-C_3_N_4_ heterojunction and Schottky barrier formed at the interfaces of Ag/TiO_2_, Ag/gC_3_N_4_ or TiO_2_/Ag/g-C_3_N_4_ (Figure 12b,c) [116,117,122,125,126]. According to this mechanism, both TiO_2_ and g-C_3_N_4_ are excited under spectrum solar irradiation, but g-C_3_N_4_ mainly absorbs visible light and TiO_2_ absorbs UV light. After excitation, photogenerated e^-^ can easily be transferred from the more negative CB of g-C_3_N_4_ to the less negative CB of TiO_2_ and, at the same time, h^+^ can be easily transferred from the more positive VB of TiO_2_ to the less positive VB of g-C_3_N_4_ (Type-II mechanism). When Ag is deposited on TiO_2_ in TiO_2_/g-C_3_N_4_, e^-^ can be transferred from the CB of TiO_2_ and trapped by Ag due to the Schottky barrier formed at the interface of the Ag and TiO_2_ (Figure 12b) [117,121,122,124,126]. This promotes the separation of charge carriers and significantly enhances the photocatalytic activity. On the other hand, when Ag is deposited on TiO_2_ and g-C_3_N_4_ in the ternary heterostructure, Ag captures the electrons from both g-C_3_N_4_ and TiO_2_ (Figure 12c) [116,117,125]. The effect of the position of the noble metal in TiO_2_/g-C_3_N_4_ on the photocatalytic activity was systematically investigated for ternary Pt/TiO_2_/g-C_3_N_4_ nanocomposites, and the results showed that the efficiency increased as follows: Pt deposited only on g-C_3_N_4_ < Pt deposited on both TiO_2_ and g-C_3_N_4_ < Pt deposited only on TiO_2_ [127].

In some of the reported studies, only visible light was used as the excitation source, and it was suggested that only g-C_3_N_4_ absorbs the visible light photons and is excited [118,120,128]. Subsequently, the photogenerated e^−^ in the CB of g-C_3_N_4_ can be transferred to the CB of TiO_2_. It has been suggested that the Ag deposited on the surface of TiO_2_ plays a key role as an electron conduction bridge, to transfer the electron from the CB of g-C_3_N_4_ to the CB of TiO_2_. The formation of a Schottky barrier at the interface between Ag and TiO_2_ efficiently enhances the electron transfer to TiO_2_ and the separation of electron−holes in g-C_3_N_4_ [118,120]. At the same time, the SPR effect of Ag contributes significantly to the absorption of visible light in the Ag/TiO_2_/g-C_3_N_4_ nanocomposite. However, it is also believed that the strong electron oscillation in the SPR in Ag under visible light triggers the transfer of energetic electrons from Ag into the TiO_2_ conduction band, thus shattering the Schottky barrier (Figure 12d) [120]. It is hypothesized that loading of TiO_2_ with Ag can cause the shift of the Fermi level of Ag to a more negative level and of TiO_2_ to a more positive level, to achieve a new Fermi level equilibrium that allows the transfer of the energetic plasmon electrons of Ag NPs across the energy barrier into the conduction band of TiO_2_ [115]. In this case, the oxidation reaction occurs at the surface of TiO_2_, while the holes of g-C_3_N_4_ are directly involved in the oxidation reaction.

In the literature, an Ag/TiO_2_/g-C_3_N_4_ ternary nanocomposite has not yet been applied to textile fibers, although it has been established as a powerful nanomaterial for the photocatalytic degradation of various dyes [43,108,113,116,118,121,123,124], phenol [118], acetaldehyde [119], formaldehyde [117], ammonia [109], and carbon dioxide [122]; hard metals, such as hexavalent chromium [108,109]; and uranium from uranium-containing wastewater [45]. Ag/TiO_2_/g-C_3_N_4_ nanocomposite has already been used for photocatalytic hydrogen evolution [115,126], solar water oxidation [112], electron transport in organic solar cells [114], vitamin B3 production [110], and as an antibacterial agent [108,111]. Due to its excellent multifunctional properties, the use of Ag/TiO_2_/g-C_3_N_4_ nanocomposite for the chemical modification of textile fibers is still a promising research challenge.

## 5. Conclusions and Future Perspectives

In this review, binary Ag/g-C_3_N_4_ and TiO_2_/g-C_3_N_4_ nanostructures and ternary Ag/TiO_2_/g-C_3_N_4_ nanostructure were presented as very promising and effective nanocomposites with Schottky, Type II, and Z-scheme mechanisms of photocatalysts. All the above nanocomposites have attracted much attention, due to their ability to initiate and carry out various reduction and oxidation reactions under visible light, and can thus be advantageous when used in environmental remediation, energy storage and conversion, sustainable catalysis, biosensing, and antimicrobial disinfection.

For textile applications, binary Ag/g-C_3_N_4_ and TiO_2_/g-C_3_N_4_ composites have emerged as promising functional nanomaterials, because the synergistic effect of the components in the heterostructures leads to improved photocatalytic performance of the composites compared with the single-component material itself. The Ag/g-C_3_N_4_ nanocomposites not only exhibit enhanced visible-light photocatalytic performance but also an improved antimicrobial performance, due to the excellent antimicrobial activity of Ag. In the preparation of Ag/g-C_3_N_4_ nanocomposites, g-C_3_N_4_ is surface-decorated with Ag during the in situ synthesis of Ag^0^ from AgNO_3_ precursor in the suspension of presynthesized g-C_3_N_4_. Another approach is to mix urea, melamine, and/or cyanuric acid with AgNO_3_ precursors and then synthesize Ag-doped g-C_3_N_4_ under suitable conditions. Compared to bare g-C_3_N_4_, it is believed that the efficiency of the Ag/g-C_3_N_4_ nanocomposite is significantly enhanced by the presence of Ag^0^, which acts as a current collector and plasmonic absorber. The Schottky barrier formed at the interface between Ag and g-C_3_N_4_ maximizes photoinduced charge carrier separation and prevents electron–hole pair recombination. The nanocomposite photoactivity is further enhanced by plasmon resonance energy transfer, as the intense electric near field induced by SPR improves the efficiency of charge carrier separation. The exceptional photocatalytic performance of Ag/g-C_3_N_4_ nanocomposites on textile substrates has already been advantageously used for the photochemical activation of organic syntheses and as a textile-based source for wearable electronics.

The two-semiconductor heterojunction of TiO_2_ and g-C_3_N_4_ has been recognized as an effective material for various environmental and energy applications. TiO_2_/g-C_3_N_4_ was prepared by various synthetic routes, including the facile mixing of prepared TiO_2_ and g-C_3_N_4_ under suitable conditions, the in situ synthesis of TiO_2_ from its precursor in the presence of presynthesized g-C_3_N_4_, or conversely, the in situ synthesis of g-C_3_N_4_ from its precursor in the presence of presynthesized TiO_2_. Moreover, the precursors of TiO_2_ and g-C_3_N_4_ were simultaneously mixed in sol to synthesize the TiO_2_/g-C_3_N_4_ heterojunction. An important step in the synthesis process is the calcination of the nanocomposites under suitable conditions, to obtain the desired morphology of the nanocomposite. The unique properties of the TiO_2_/g-C_3_N_4_ heterojunction are related to the simultaneous utilization of UV and visible light, resulting in excellent photocatalytic performance under UV- and visible-light irradiation. When TiO_2_/g-C_3_N_4_ is excited by incident UV/visible light of sufficient energy, the Type-II heterojunction and the direct Z-scheme charge carrier transfer pathway are adopted in the photocatalytic mechanism of the TiO_2_/g-C_3_N_4_ nanocomposite. According to the band edge potentials, the Type-II heterojunction allows the transfer of the photogenerated electrons from the CB of g-C_3_N_4_ to the CB of TiO_2_ and the photogenerated holes from the VB of TiO_2_ to the VB of g-C_3_N_4_. This causes the reduction reaction on TiO_2_ to proceed with a lower reduction potential compared with g-C_3_N_4_, and the oxidation reaction on g-C_3_N_4_ to proceed with a lower oxidation potential compared to TiO_2_, which is a disadvantage of the Type-II heterojunction mechanism. On the other hand, despite having the same band structure configuration, the direct Z-scheme assumes a much stronger redox capability for the TiO_2_/g-C_3_N_4_ heterojunction because it promotes the spatial separation of electrons and holes on g-C_3_N_4_ with the higher reduction potential and on TiO_2_ with the higher oxidation potential, respectively, and promotes the recombination between the photogenerated electrons in the CB of TiO_2_ and the photogenerated holes in the VB of g-C_3_N_4_ with the lower redox capability. The superior photocatalytic efficiency of TiO_2_/g-C_3_N_4_ nanocomposite has already been beneficially utilized in the development of textile-based photocatalysts for the effective purification of liquid and gaseous pollutants and bacteria.

There is no evidence in the literature that an Ag/TiO_2_/g-C_3_N_4_ ternary nanocomposite has been used for textile applications, although it is a promising strategy for the surface- and bulk-modification of textiles. There are many strategies to prepare Ag/TiO_2_/g-C_3_N_4_ heterostructures, including simultaneous synthesis of the nanocomposite from suitable precursors, in situ synthesis of Ag in the presence of a previously synthesized TiO_2_/g-C_3_N_4_ composite, and surface decoration of TiO_2_ by Ag and subsequent mixing with g-C_3_N_4_. The synthesis pathway directly affects the photocatalytic mechanism of the Ag/TiO_2_/g-C_3_N_4_ nanocomposite, which can be explained by the direct Z-scheme or the Type-II mechanisms established in the TiO_2_ and g-C_3_N_4_ heterojunction, supported by the Schottky barrier and SPR of Ag. The great potential of the Ag/TiO_2_/g-C_3_N_4_ ternary nanocomposite lies in its ability to provide multifunctional textile properties, such as photocatalytic self-cleaning, antimicrobial activity, UV protection, conductivity, and thermal stability. Therefore, the development of textile platforms with integrated Ag/TiO_2_/g-C_3_N_4_ heterostructures is a major challenge, where the in situ synthesis of Ag/TiO_2_/g-C_3_N_4_ in the presence of textile fibers as an stabilizing agent is a priority. Due to the additional requirements imposed on textile materials, the preparation of simultaneously effective, multifunctional, non-cytotoxic, and durable chemical modification of textile substrates will certainly be a hot research topic and will open new application opportunities for textile-based Ag/TiO_2_/g-C_3_N_4_ nanocomposites.

## Data Availability

The data presented in this study are available on request from the corresponding author.
